# Incidental lung mass: a rare case of intrapulmonary schwannoma mimicking a pulmonary hydatid cyst

**DOI:** 10.1093/omcr/omaf306

**Published:** 2026-02-18

**Authors:** Milan Pokhrel, Bibek Shrestha, Bandana Ghimire, Ayusha Gautam, Simran Rauniyar, Sonam Dhenga

**Affiliations:** Department of Internal Medicine, Maharajgunj Medical Campus, Institute of Medicine, Tribhuvan University, Kathmandu, Bagmati State 44600, Nepal; Department of Internal Medicine, Maharajgunj Medical Campus, Institute of Medicine, Tribhuvan University, Kathmandu, Bagmati State 44600, Nepal; Department of Internal Medicine, Maharajgunj Medical Campus, Institute of Medicine, Tribhuvan University, Kathmandu, Bagmati State 44600, Nepal; Department of Pathology, Maharajgunj Medical Campus, Institute of Medicine, Tribhuvan University, Kathmandu, Bagmati State 44600, Nepal; Department of Internal Medicine, Maharajgunj Medical Campus, Institute of Medicine, Tribhuvan University, Kathmandu, Bagmati State 44600, Nepal; Department of Internal Medicine, Maharajgunj Medical Campus, Institute of Medicine, Tribhuvan University, Kathmandu, Bagmati State 44600, Nepal

**Keywords:** schwannoma, hydatid cyst, pulmonary, histopathology

## Abstract

Schwannomas are rare, mostly asymptomatic neoplasms originating from peripheral nerves, with primary occurrence in the lung being exceptionally rare. Diagnosis is primarily incidental or prompted by symptoms caused by mass effect on nearby organs. Lacking characteristic clinical or radiological features, their appearance as cystic lung lesions are very likely to be confused with pulmonary hydatidosis, especially in the endemic regions. Thus, whenever an individual presents with an incidental lung mass, schwannoma needs to be considered as one of the differentials alongside other benign cystic lesions or neoplasms.

## Introduction

Schwannomas are rare, benign, slow-growing neoplasms arising from Schwann cells of peripheral nerves, the spinal cord, or cranial nerves, usually affecting males in their 3^rd^ -4^th^ decade. Schwannomas commonly occur in the head, neck, limbs, mediastinum, or retroperitoneum, with cranial nerve VIII being the most common site; however, they may affect any peripheral or cranial nerve [1, 2]. Lung parenchyma or pleura are extremely rare locations for this tumor, even more so as a primary lesion. [2, 3] They often go undiagnosed due to their sporadic, indolent and predominantly asymptomatic nature, often detected incidentally on thoracic imaging performed for unrelated reasons [1, 2]. Hydatid disease, also known as hydatidosis or echinococcosis, is an anthropozoonosis caused by Echinococcus, which may affect any part of the body, but mostly affects the liver (75%) and the lungs (15%) by forming cystic lesions. Complicated and atypical forms are hard to detect both radiologically and clinically, as they are often mistaken for other pulmonary lesions, mainly lung malignancies [4, 5].

## Case report

A 63-year-old female with 40 pack years of smoking presented to the surgical outpatient with intermittent, non-progressive pain in the epigastric region aggravated by food intake, mainly fatty food, for 2 years, associated with vomiting without any history suggestive of UGI bleed. There was no history of cough, chest pain, difficulty breathing, or fever. However, she complained of weight loss and loss of appetite. General and systemic examination was unremarkable. Ultrasonography of the abdomen was done, revealing gallstones, and the patient was planned for Laparoscopic cholecystectomy.

**Figure 1:**
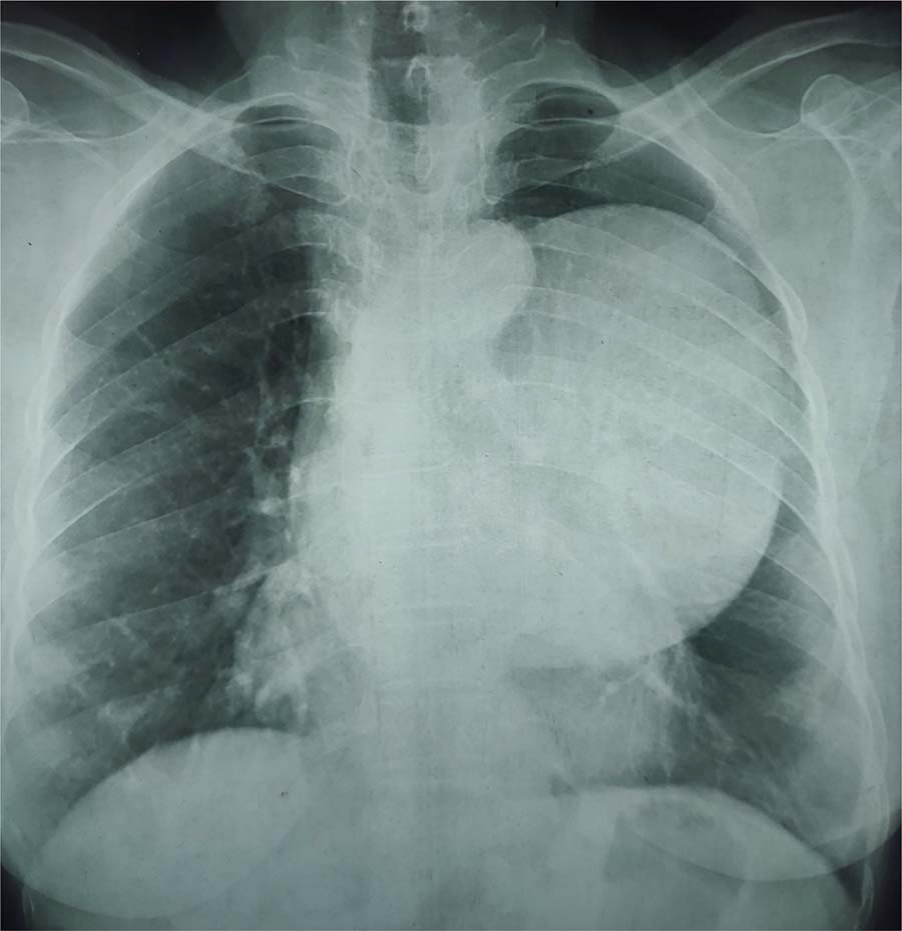
Chest X-ray (PA view) showing large, well-defined homogenous opacity in the left middle lung zone, causing silhouetting of the left hemidiaphragm and cardiac border.

**Figure 2:**
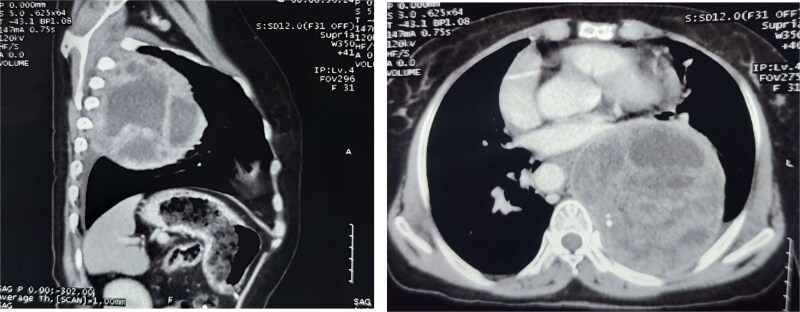
CT chest (axial and sagittal views) showing a large, multiloculated cystic lesion in the left lower lobe, causing compression of adjacent lung parenchyma and mediastinal structures.

**Figure 3:**
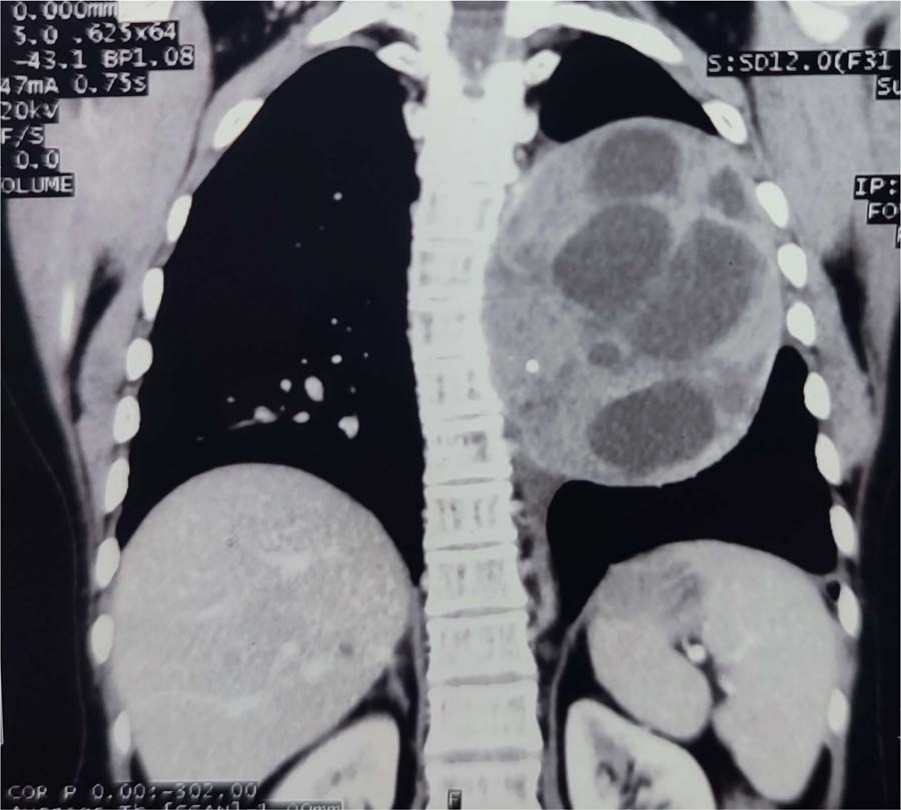
Contrast-enhanced CT of the chest demonstrates a large, well-defined, multiloculated cystic lesion in the left lower lobe of the lung, measuring approximately 11.2 × 9.8 × 11.6 cm. The lesion contains multiple internal septations and daughter cysts with areas of calcification. It causes mass effect, displacing the left main pulmonary artery, left main bronchus, descending aorta, and esophagus.

**Figure 4:**
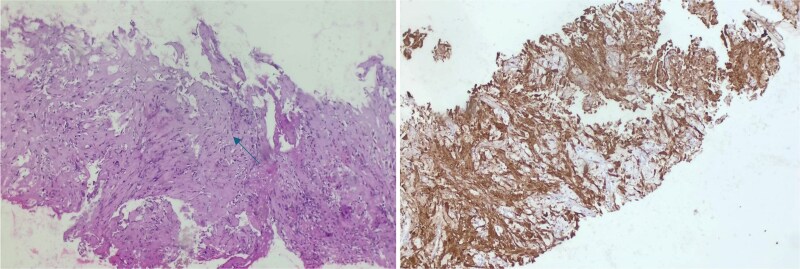
Histopathological examination (left side image) of the lung biopsy showing spindle-shaped tumor cells arranged in fascicles with alternating hypercellular (Antoni a) and hypocellular (Antoni B) areas on Hematoxylin and eosin stain (H&E, 200×). Immunohistochemical staining (right side image) demonstrating strong nuclear and cytoplasmic positivity for S-100 protein, confirming the diagnosis of schwannoma (IHC, 200×).

Upon sending preoperative investigations, the Chest X-ray showed a round heterogeneous opacity measuring around 10 cm in the left lower zone (Fig. 1). Following this pulmonology consultation was done. A CT chest was ordered, which demonstrated a round cystic lesion in the left lower lobe with enhancing septations, multiple cysts, and foci of calcifications within it abutting and displacing the left main pulmonary artery and left main bronchus anteriorly. There was a mild left pleural effusion and pericardial effusion. These features were suggestive of a hydatid cyst with abscess being a differential diagnosis (Figs 2, 3, 4) In view of the endemicity of hydatid disease in our part of the world, along with supportive radiological findings, a diagnosis of hydatid cyst was established. Echinococcus serology was sent. Meanwhile, antihelminthic medication was started. The patient did not respond to the medication, and the serology came back negative. Then, a USG-guided biopsy of the lung mass was done, and histopathological examination revealed a pulmonary schwannoma. The patient was managed conservatively and advised to follow up after 1 month with pulmonology as well as thoracic surgery OPD.

## Discussion

Various lung tumors (such as Mucinous cystic tumors [6], pseudomyxoma pleurii [7], cystic teratomas [8], lung carcinoma [9]) and benign cystic lesions of the lung (such as bronchogenic cyst, congenital adenomatoid malformation, hydatid cyst or abscess) have been reported to mimic each other radiologically as a cystic lesion [6]. Occasionally, various conditions like thoracic empyema, mediastinal mass, tuberculosis pleurisy, thoracic wall tumor, hamartomas, leiomyoma, etc have been reported to be mistaken for intrathoracic hydatidosis mainly in endemic regions [4, 10, 11]. Lung Schwannoma as its mimic has been reported once in the world literature [12]. All these uncommon yet clinically imperative mimics, which demand careful consideration, have often been misinterpreted as pulmonary hydatidosis, more so in endemic regions owing to their overlapping radiological features and inconsistent clinical and laboratory findings. Thoracic schwannoma shows few or no symptoms at all, with slow growth and gradual progression, especially in the early stages. Lesions as enormous as 10 cm are incidentally discovered. Exceptionally, some large tumors may cause pain or neurological symptoms due to mass effect on nearby structures and may manifest as tracheal deviation, dysphagia, dyspnea, dysphonia, or stridor. Sometimes individuals can present with more severe signs, such as hemoptysis when the tracheobronchial tree is involved, or gastrointestinal bleeding in cases affecting the esophagus [12, 13]. Pulmonary hydatid cysts are classically solitary and unilateral [5]. Non-ruptured pulmonary hydatid cysts are mostly found incidentally; however variable symptoms may appear based on the location and compression of adjacent structures mainly after cyst reaches 5 cm or more. A ruptured or complicated cyst may exhibit symptoms such as productive cough, expectoration of cystic contents, hemoptysis, fever, shivers, and chest pain [4, 5].

Serological tests, though helpful, lack specificity [5]. In the context of an aberrant clinical picture and unremarkable laboratory tests, radiological scans gain importance in the diagnosis stage of schwannoma, although they are relatively nonspecific [2, 13, 14]. Typically, they are solid, solitary, well-circumscribed tumours on imaging [14]. Uncomplicated cysts appear as well-defined, round or homogeneous masses, with central fluid density [9]. Lung atelactasis, mediastinal shift, or pleural effusion may be the findings in larger cysts, depending on pressure on the neighbouring tissue [4]. Evaluation of complicated, atypical, ruptured, or infected forms is often difficult because the infiltration and infection of surrounding parenchyma may give a soft tissue aspect, making them resemble lung malignancies or abscesses in a CT scan [4, 5, 9]. Thus, definitive diagnosis requires histopathological confirmation. Microscopically, these tumors have two distinct growth patterns: Antoni A and Antoni B, representing areas of hyper and hypocellularity, respectively. Historically, surgical resection has been the primary treatment approach, typically involving removal of the affected lung lobe via thoracoscopy or thoracotomy or complete pleural resection with frequent follow-ups [3, 14]. With the advancement of bronchoscopic technology, less invasive approaches (including cryotherapy, high-frequency electrocoagulation, and laser therapy) have emerged, particularly for patients with surgical risks or those preferring non-surgical interventions [3]. Tumours can also be left untreated and monitored due to their low malignant potential. Indications for surgery are predominantly symptoms caused by mass effect, including pain, neurological and respiratory symptoms, or diagnostic uncertainty. Thoracic Schwannomas are generally considered to have an excellent prognosis due to effective therapeutic options and minimal risk of distant spread [2]. This case emphasizes the critical need to include lung schwannoma in the differential diagnosis of pulmonary hydatid cyst in endemic regions, particularly when clinical, radiological, and serological profiles are overlapping and misleading. Diagnosis in such cases should usually be made on histopathological examination of surgical biopsy/resected specimens.
